# Tegmen Tympani Defect and Brain Herniation Secondary to Mastoid Surgery: Case Presentation

**DOI:** 10.1155/2014/756280

**Published:** 2014-07-21

**Authors:** Oguz Kadir Egilmez, Fatih Mehmet Hanege, M. Tayyar Kalcioglu, Tuncay Kaner, Numan Kokten

**Affiliations:** ^1^Department of Otorhinolaryngology, Goztepe Training and Research Hospital, School of Medicine, Istanbul Medeniyet University, 34722 Istanbul, Turkey; ^2^Department of Neurosurgery, Goztepe Training and Research Hospital, Istanbul Medeniyet University School of Medicine, 34722 Istanbul, Turkey

## Abstract

Brain herniation into the middle ear is very rarely seen. In addition to reasons like congenital factors, trauma, and infection, tegmen defect may develop as a result of iatrogenic events secondary to chronic otitis media surgery with or without cholesteatoma. Since it may cause life-threatening complications, patients must be evaluated and monitored for tegmen defect. In this paper, diagnosis and treatment of a brain herniation case due to iatrogenic tegmen defect were described along with relevant literature.

## 1. Introduction

Brain tissue herniation through tegmen tympani defect is rare in otorhinolaryngology practice. Although it mostly depends on congenital skull base defects, trauma, infection, and tumours, idiopathic and iatrogenic cases are also encountered [1–4]. They are usually observed in patients who underwent mastoid cavity surgery due to chronic otitis media with or without cholesteatoma [5, 6]. The incidence of encephalocele developed due to tegmen defect is decreased with the use of broad-spectrum antibiotics and technological developments in ear surgeries [5]. Although there is no standard surgery protocol, three approaches are generally acceptable: middle fossa approach, transmastoid approach, and combination of both [3, 5]. In this case presentation, diagnosis and treatment of brain herniation through tegmen defect developed after mastoid surgery are discussed along with relevant literature.

## 2. Case Presentation

**Figure 1:**
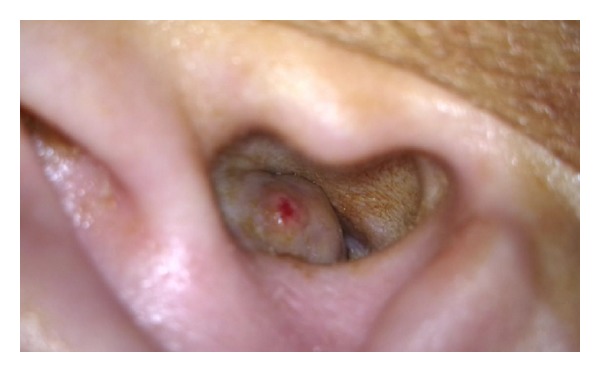
A soft mass-like lesion is at the posterosuperior wall of right external auditory canal entrance.

**Figure 2:**
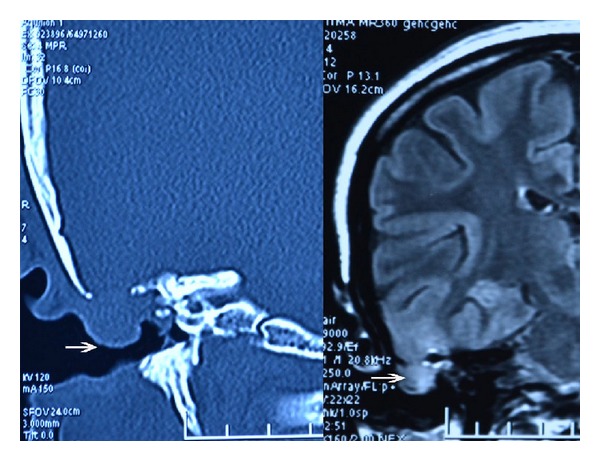
MRI and CT images of the tegmen defect and herniated brain tissue.

**Figure 3:**
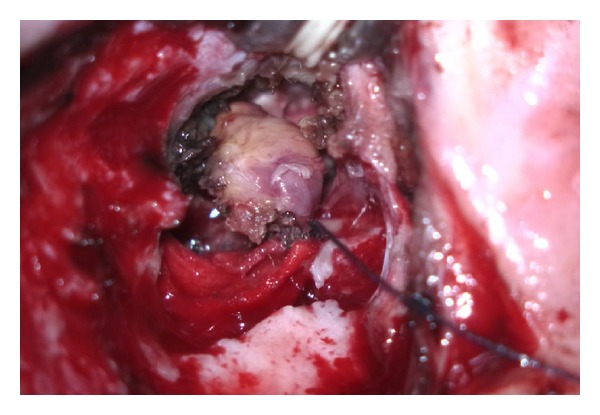
Herniated fibrotic glial tissue is excised by neurosurgery team.

**Figure 4:**
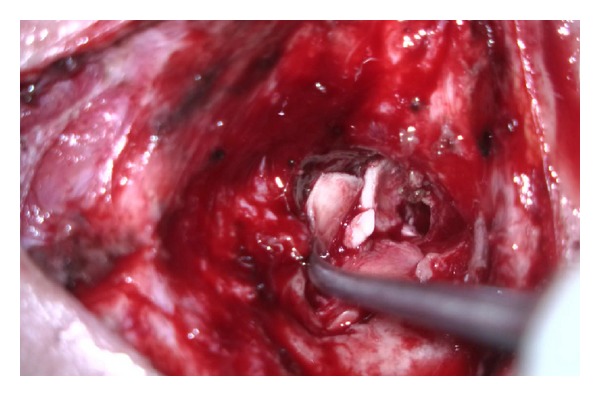
Cavity is obliterated with cartilage grafts.

**Figure 5:**
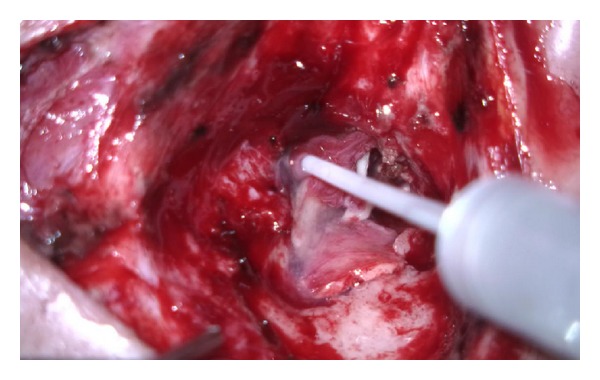
Durability is maintained by fibrin glue (Tisseel Kit).

Admitted to our clinic with complaints of right ear discharge from childhood, hearing impairment, occasional attacks of dizziness, and pain in face and ear; a 33-year-old woman who underwent radical mastoidectomy for chronic otitis media with cholesteatoma at an external facility on May 2012. A growing mass was observed about 2 months postoperatively at the entrance of the right external auditory canal. Patient had no complaints such as ear discharge, dizziness, or epilepsy. Patient applied to our clinic at about 10 months postoperatively. Her medical examination showed a soft mass-like lesion with a diameter of about 1.5 cm at the posterosuperior wall of the right external auditory canal entrance (Figure 1). Temporal bone computed tomography and cranial contrast MRI were performed. CT showed a 13 mm defect at tegmen of right temporal bone. MRI showed a 12 mm defect at lateral segment of right tegmen tympani and a nodular signal of about 15 × 13 × 10 mm, which was isointense to brain parenchyma in all sequences filling the right middle ear and proximal segment lodge of the external ear canal (Figure 2). The mass was determined to be dural prolapsus and focal herniation of brain parenchyma. Neurosurgery clinic was consulted and surgery was scheduled. The operation was accompanied by neurosurgeons. After ensuring the operation site through transmastoid approach under general anaesthesia, patient was referred to neurosurgery team. Neurosurgery team excised the herniated fibrotic glial tissue (Figure 3) extending to external ear canal by using bipolar cautery. Duraplasty with galea was performed after determining bone borders. A barrier was created with fibrin glue (Tisseel Kit). Because of the absence of perioperative CSF, case was referred to otorhinolaryngology team once more. Grafts were taken from conchal cartilage and temporalis muscle fascia. Cavity was obliterated with cartilage grafts (Figure 4). A flat plane was created by laying the temporalis muscle fascia graft on cartilage grafts. Durability was improved with fibrin glue (Tisseel Kit) (Figure 5), and thus the operation was completed. There were no complications such as postoperative otorrhoea, meningitis, or epilepsy. Patient had no complaints in followups at week 1 and months 1, 3, 6, and 15 postoperatively and no pathology was observed at the herniated segment through the defective area.

## 3. Discussion

Bone erosion and dural injury can be observed due to chronic suppuration or as a complication of mastoid surgery in chronic otitis media [2]. In many cases, it is difficult to determine whether the defect is iatrogenic or a result of suppuration. There are two explanations of the mechanism of bone defect caused by cholesteatoma: first, occurrence of bone resorption due to cholesteatoma, which causes local ischemia as a result of its pressure on bone wall, and, second, occurrence of bone erosion with the involvement of inflammatory process due to enzymatic destruction [7]. Defects were observed to be located at anterosuperior portion of petrous bone in 64% of iatrogenic-origin cases [1]. Also in this case, defect was detected at anterosuperior portion of petrous bone and in addition, a mass was observed to develop at external ear canal at two months postoperatively. These findings support the notion that etiology of this defect is iatrogenic.

Usually, three different surgical approaches are used in cases of meningoencephalocele due to tegmen defect: transmastoid approach, middle fossa approach, and combination of both [8]. For lateral and posterior defects of tegmen (tegmen antri) or defects which are smaller than 1 cm, the transmastoid approach may be the favorable choice [9]. A middle cranial fossa approach is the most appropriate for anterior and medial defects of the tegmen (tegmen tympani) or large defects over 2 cm or multiple defects [10, 11]. Musculus temporalis fascia and cartilage may be used alone as repair techniques or the defect may also be supported with materials such as muscle, bone, and fibrin glue [12]. Selection of these materials depends on the experience of the surgeon, size of defect, and volume of herniated brain tissue [5]. Mostly, conchal cartilage is preferred because of its ease of use, curled shape, and easy moulding resembling the middle cranial fossa base [3]. In our case, transmastoid approach was chosen by considering the localization and the size (12 mm) of the defect. Dura was secured with fibrin glue after removal of fibrotic glial tissue and defect was closed with combination of conchal cartilage graft and fascia graft.

Postoperative complications such as epileptic seizures, CSF leak, transient ischemic attack/stroke, sepsis, and sensorineural hearing loss may be encountered after repairment [12]. However, these complications were not observed in our case neither in early nor in late followups.

## 4. Conclusion

All patients who underwent operation due to chronic otitis media with or without cholesteatoma must be evaluated for tegmen defect and brain tissue or dural structures that may be herniated through this defect during and after the surgery. Possible defects must be repaired with appropriate surgery methods and graft materials by considering the localization and the size of the defected area.
